# Thymoquinone effect on the *Dictyostelium discoideum* model correlates with functional roles for glutathione S-transferases in eukaryotic proliferation, chemotaxis, and development

**DOI:** 10.1371/journal.pone.0282399

**Published:** 2023-03-01

**Authors:** Nida Alsaffar, Yayin Fang, Eric Walters

**Affiliations:** Department of Biochemistry and Molecular Biology, Howard University College of Medicine, Washington, DC, United States of America; Texas A&M University College Station: Texas A&M University, UNITED STATES

## Abstract

An increasing body of literature demonstrates the therapeutic relevance of polyphenols in eukaryotic cell and animal model studies. The phase II glutathione S-transferases (GST) show differential responses to thymoquinone, a major bioactive polyphenol constituent of the black seed, *Nigella sativa*. Beyond antioxidant defense, GSTs may act in non-enzymatic capacities to effect cell cycle, motility, and differentiation. Here, we report the impact of thymoquinone on the life cycle of the eukaryotic model *Dictyostelium discoideum and* accompanying profiles of its GST-alpha (DdGSTA) enzyme activity and isozyme expression. *In silico* molecular modeling revealed strong interaction(s) between thymoquinone and DdGSTA2 and DdGSTA3 isozymes that correlated with *in vivo*, dose-dependent inhibition of cell proliferation of amoebae at 24, 48, and 72hr. Similarly, cytosolic DdGST enzyme activity (CDNB activity) was also responsive to different thymoquinone concentrations. Thymoquinone generally reduced expression of DdGSTA2 and DdGSTA3 isozymes in proliferating cells, however differential expression of the isozymes occurred during starvation. Thymoquinone effectively reduced early-stage aggregation of starved amoeba, accompanied by increased reactive oxygen species and altered expression of tubulin and contact site A (gp80), which resulted in reduced morphogenesis and fruiting body formation. These observations reveal that thymoquinone can impact signaling mechanisms that regulate proliferation and development in *D*. *discoideum*.

## Introduction

Glutathione S-transferase (GSTs) enzymes metabolize many substrates by the conjugation of glutathione (γ-L-glutamyl-L-cysteinyl-glycine, GSH) to hydrophobic and electrophilic molecules, rendering them less toxic and subject to further modification or cellular elimination [1, 2]. Despite their predominant role in detoxification, GSTs contribute to other eukaryotic cellular processes, including proliferation, motility, and homeostasis. However, their direct action(s) in the regulation of these events across eukaryotes is poorly understood. It is well known that pharmacologic induction and inhibition of GST isozymes in eukaryotic cells can alter cellular homeostasis and signaling that underlies cell proliferation, development, differentiation- [3, 4]. Polyphenols are known to influence GST expression and activity, leading to changes in signaling, amid local and systemic function of cells, organs, and tissues. In this regard, the use of medicinal polyphenol plant extracts for therapeutic prophylaxis, intervention, and treatment of disease is of interest [5]. *D*. *discoideum* expresses five GSTs isozymes (DdGSTα1-α5 class), however less is understood regarding their functional roles in the programs of development and morphogenesis of the organism. *D*. *discoideum* grows as a unicellular amoeba and feeds on readily available nutrients and bacteria. The depletion of its food source initiates a program of cellular chemotaxis, multicellular differentiation, and morphogenesis. As such, the *D*. *discoideum* eukaryote exhibits stage-specific features with distinct biochemical, molecular, and cellular signatures that can be exploited to identify specific molecular effect(s) [5, 6] of drugs, chemicals, and biological factors on its physiology, growth, and development. Specifically, the *D*. *discoideum* model has been useful to characterize the intracellular targets of bisphosphonates [7], lithium drugs [8], green tea [9], turmeric-derived curcumin [10–12], and naringenin [13]. Our laboratory has reported that curcumin, which is currently employed in a number of clinical trials, alters DdGST enzymatic expression and activity, and interferes with proliferation, chemotaxis, and development [10].

Thymoquinone (2-isopropyl-5-methylbenzo-1, 4-quinone; C_10_H_12_O_2_) [14, 15], a predominant bioactive polyphenol constituent of the black seed *Nigella sativa*, exhibits pharmacological potency against several diseases [15–18]. Thymoquinone possesses significant therapeutic efficacy, harboring anti-cancerous, anti-proliferative, and anti-inflammatory potential either as an individual or combined treatment modality [19]. Indeed, thymoquinone is widely used traditionally and currently employed in clinical trials for the treatment/prevention of multiple types of cancer, showing a potential to mitigate components of tissue microenvironments at several stages by various pathways [20]. The use of therapeutic substances like thymoquinone for the treatment of diseases and biomedical research necessitates the identification of their molecular targets and actions at cellular and sub-cellular levels. The life cycle of *D*. *discoideum* involves stages of single cell autonomy, chemotactic aggregation, multicellular differentiation, and morphogenesis. Exploring the impact of thymoquinone on biochemical targets at various stages of *D*. *discoideum* development can lead to further insight regarding its impact on unicellular and multicellular mechanisms that parallel tissue microenvironments in mammalian cells. In this study, molecular modeling indicated strong interactions between thymoquinone and DdGSTA isozymes, which prompted us to characterize the effect of thymoquinone on DdGSTs *in vivo*, and its influence on proliferation, growth, and development of the organism. We report that thymoquinone exerts generally negative effects on DdGSTA enzymes, proliferation, and development of *D*. *discoideum*. These findings suggest thymoquinone directly binds to GSTs, and likely interrupts key events in early *D*. *discoideum* YakA signaling and morphogenesis. This research further illuminates the role of GSH-mediated events that govern basic cellular programs within eukaryotic cell-cell interaction and tissue homeostasis.

## Materials and methods

### *In-silico* molecular interactions

Molecular interactions of the amino acid residues of the DdGSTs with thymoquinone were investigated through *in-silico* study. The 3D structures of target protein DdGSTA2, DdGSTA3, were obtained from PDB database (http://www.rcsb.org/) [21]. The ligand (thymoquinone) was generated by using builder module of the Molecular Operating Environment (MOE) [22]. Subsequently, all possible binding sites in each target were predicted by the using Site Finder module of MOE and the docking scores of the ligand (thymoquinone) with each possible site were collected. The site in a target with highest docking score was identified as its putative active site and the molecular interactions between the target protein and ligand in the docked complex were identified for the analysis [23].

### *Dictyostelium discoideum* growth and proliferation

*Dictyostelium* AX4 (axenic) amoebae were grown at 22°C on a rotary shaker (180 rpm) in HL5 media containing 100 mg/mL of streptavidin and 100 units/mL penicillin. Initially, the IC50 for thymoquinone was determined by seeding amoebae at 5x 10^4^ cells/ml with 0.25, 0.5, 1, 2.5, 5.0, 7.5, and 10 μM) for 0, 24, 48, and 72 hours to determine working concentrations of thymoquinone. Viability of amoeba was determined by Trypan blue stating of the amoeba for all time points. For subsequent proliferation studies, thymoquinone was prepared in HL5 and added to 5x 10^4^ cells/mL at 0, 2.5, 5, and 7.5μM. Cell counts and trypan blue staining for viability was obtained by hemocytometer at 24, 48, and 72hr in accordance with previously published studies [10].

### DdGSTs enzyme activity assay (CDNB assay)

Amoebae (5x 10^4^ cells/mL) were washed in PBS and sonicated on ice using three 10-second bursts at high intensity (Heat Systems Ultrasonic Cell Disruptor) and cooling for 30 seconds on ice between each burst. Protein concentrations were estimated by the method of Bradford using bovine serum albumin as the standard [24]. DdGSTs activity was determined at 25°C with reduced glutathione (GSH) and 1-chloro-2, 4-dinitrobenzene (CDNB) as substrates, measuring the increase in spectrophotometric absorbance at 340nm for 5 minutes [25]. CDNB activity assays of purified, recombinant GSTs (rDdGSTA2, rDdGSTA3) were conducted in accordance with protocols previously established in our laboratory [26]. Data presented as mean enzyme activity (% control) ± SEM. Statistical significance was determined using the one-sample t-test (mean, 100; two-tailed) vs. control, *p < 0.05.

### Starvation-induced aggregation and agar development

For aggregation studies, axenically grown cells were washed three times in 1X KK2 buffer (20 mM potassium phosphate, 2 mM magnesium chloride, 0.1 mM calcium chloride, pH 6.1) and resuspended in 1X KK2 at 1 x 10^6^ cells/mL in the absence or presence or thymoquinone (2.5, 5.0, 7.5uM) and placed on a rotational shaker (110 rpm). At 5–6 hr, 10 uL of the cell aggregates was observed under a hemocytometer. Images of aggregates (n = 5/controls and treatment groups) were captured on a Nikon ES400 microscope, and their two-dimensional areas were quantified using ImageJ 1.54b software; one-way ANOVA was used to determine statistical significance.

For development studies, axenically grown cells were washed three times in 1X KK2 buffer (20 mM potassium phosphate, 2 mM magnesium chloride, 0.1 mM calcium chloride, pH 6.1) and 1 x 10^7^ cells/mL and 10uL of the suspension was spotted onto non-nutrient agar plates that contained 0, 2.5, 5.0, and 7.5 μM thymoquinone. Development and morphogenesis were monitored during a 40hr period.

### Gel electrophoresis/western blotting

Amoebae lysed in RIPA buffer containing protease inhibitors (Sigma), were sonicated, and centrifuged at 14,000 *rpm* for 20 min at 4°C. Supernatant protein concentrations were calculated, and samples electrophoresed on 10% SDS-polyacrylamide gels. Proteins were electroblotted onto PVDF membranes (Millipore) and blocked in PBST (PBS containing 0.1% Tween 20)/5% milk. Membranes were incubated with antibodies directed against DdGSTA2 or DdGSTA3 [10] (1:2000 each), discoidin I, alpha-tubulin, and csA/gp80 proteins (0.5ug/mL each, obtained from the Developmental Hybridoma Bank). Immunoblots were washed in PBST (PBS/0.1% Tween 20) were incubated with species-specific peroxidase-labeled secondary antibodies, developed either by chemiluminescent or colorimetric (3–3, diamino benzidine) methods. Blots were scanned or imaged using a BioRad Versadoc 3000 system, and densitometry and quantitation was performed with ImageJ software.

### Enzyme-linked immunosorbent assay (ELISA)

The quantitation of DdGSTA2 and DdGSTA3 isozymes was in accordance with established protocols [27]. Samples containing 100ng of extract were used to coat 96-well polystyrene microplates and incubated for 2h. The wells were washed with PBS including 0.05% Tween-20 (PBST) and incubated for 2h with blocking buffer (10 mM sodium phosphate, pH 7.3, 2% bovine serum albumin (BSA). After washing, anti-DdGSTA2 and DdGSTA3 antisera (1:1000) in blocking buffer was added to each well and incubated for 2h at 37°C. Wells were washed with PBST and incubated with horseradish peroxidase (HRP)-conjugated goat anti-rabbit (DdGSTA2) or donkey anti-chicken (DdGSTA3) IgG (1:3000 each), for 1h at 37°C. After three washes, 3,3,5,5 -tetramethylbenzidine (TMB) substrate was added and incubated for 10 minutes, and development was terminated with 1N sulfuric acid, and samples read at 450nm.

### Measurement of ROS

The production of ROS was measured using DCFDA (2’-7’dichlorofluorescin diacetate) dye (1μg/ml, Sigma). Control and treated *D*. *discoideum* amoebae (1×10^6^ cells/ml) were starved in 1X KK2 buffer in the presence of thymoquinone for six hours, then gently washed with PBS followed by the addition of DCFDA, and incubated for 30 min at 22°C with gentle shaking. DAPI was added to aggregated cells and fluorescence was captured using a Nikon ES400 microscope [28]. Quantitation of DCFDA staining was performed by measuring intensity values of comparable sized clusters of aggregated cells for three independent samples and performing statistical evaluation of relative intensity using averages of five clusters/group, followed by the Student’s t-test.

### Quantitation of GSH

The quantification of reduced (GSH) and oxidized (GSSG) levels was adapted from previously published studies [29, 30]. In brief, amoebae were starved in 1X KK2 buffer in the presence or absence of thymoquinone at various concentrations for 6hr, then harvested, and immediately deproteinized using 5% sulfosalicylic acid. Samples were freeze-thawed twice, centrifuged, and the supernatant used for GSH quantitation based on thiol reagent DTNB (5-5-dithiobis (2-nitrobenzoic acid), with the 3,3′,5,5′-tetramethylbenzidine (TMB) colorimetric development detected spectrophotometrically at 412 nm. A microplate assay system was used to determine GSH/GSSG ratios on three independent samples/group.

### Statistical analysis

Where indicated, results are expressed as mean values ± the standard error, and statistically analyzed with GraphPad Prism 4.0 version (GraphPad Prism Software, Inc. San Diego, CA, USA). The Student’s t-test or one-way ANOVA was used to determine significance.

## Results

### *In silico* molecular interactions, thymoquinone and DdGSTA2, DdGSTA3 isozymes

**Fig 1:**
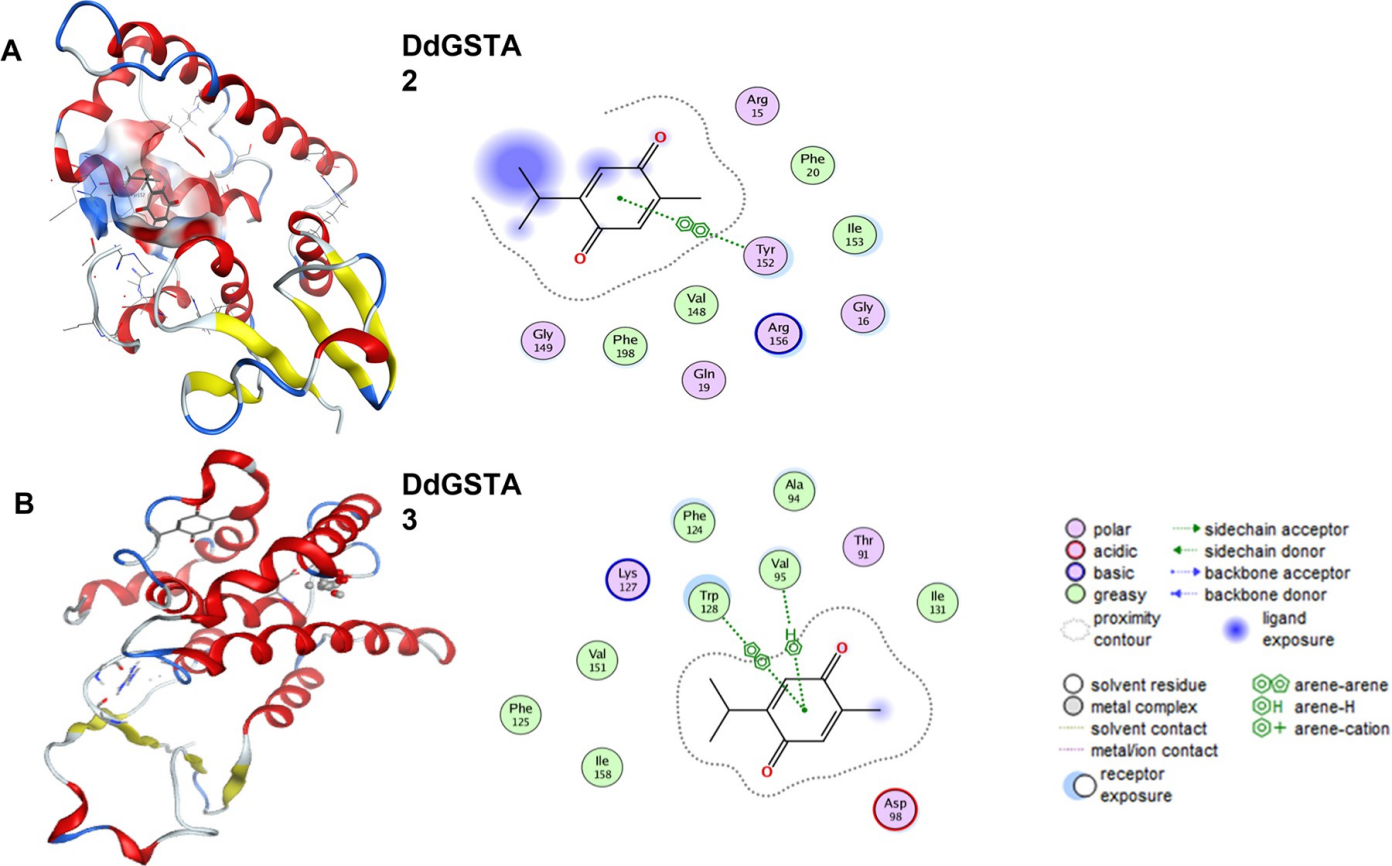
Computational model showing the binding interactions of thymoquinone (TQ) with DdGSTA2 and DdGSTA3. A and B: DdGSTA2 and DdGSTA3 isozymes and corresponding 2D diagrams of the ligand interaction map for TQ. Interactions with TQ illustrate hydrogen-bonding or electrostatic interaction at the binding pocket. For DdGSTA2, the 2D interaction map contain contact residue in the pocket and details of one π-π interaction is possible with Tyr152. For DdGSTA3, the 2D interaction map contain contact residue in the pocket and details of a π-H interaction is possible with amino acid Val95 and a π-π interaction (π-π) is possible with Trp128.

Recent findings in our laboratory reveal that *D*. *discoideum gstA2* and *gstA3* transcription profiles are responsive to curcumin polyphenol [10]. Additional evidence suggests that the DdGSTA2 and DdGSTA3 isozymes may play important roles in *Dictyostelium* growth and development [26]. To examine the interaction between thymoquinone and individual isozymes of DdGSTA2 DdGSTA3, we performed *in silico* docking analyses. Findings revealed that thymoquinone interacts with DdGSTA2 and DdGSTA3 at multiple sites. Stabilized pockets of thymoquinone-GST presented docking scores of -5.0005 kcal/mol and -4.5216 kcal/mol for the DdGSTA2 and DdGSTA3 isozymes, respectively, illustrated in (Fig 1A and 1B). Regarding DdGSTA2, ARG15 PHE150 ASP151 TYR152 ARG154 PHE155 ARG156 LYS177 ILE190 LYS191 GLU192 ARG193 PRO194 GLU195 THR196 LYS197 represent the top pocket amongst 18 possible binding sites in isozyme that interact with thymoquinone. Thymoquinone interaction with DdGSTA3 shows that THR91 ALA94 VAL95 ASP98 PHE124 LYS127 TRP128 ILE131 LEU32 ALA139 represent the top-ranking sites (Fig 1B). Of the 16 amino acids in the DdGSTA2 isozyme that bind to the ligand, one π-π interaction is possible at TYR152, which is essential for substrate binding at the H-site. In contrast, of 10 amino acids that bind thymoquinone to DdGSTA3, one π-π interaction (TRP128) and another π-H interaction (VAL95) are present (Table 1). Additional information can be found in S1 Data.

**Table 1:** NCBI binding domain of DdGSTA2 and DdGSTA3 amino acid sequences and thymoquinone in silico docking score analysis.

GST Isozymes	Protein ID	Thymoquinone Docking Score	Thymoquinone Pocket (Amino Acid Binding)	DdGST “H” Site Residues for Substrate
DdGSTA2	Q556G3	-5.0005	Arg15 **Gly149** Phe150 Asp151 **Tyr 152** Arg154 Phe155 Arg156 Lys177 Ile190 Lys191 Glu192 Arg193 Pro194 Glu195 Thr196 Lys197	His98 Ala99 Phe102 pro103 **Gly149 Try152**
DdGSTA3	Q54VI4	-4.5216	Thr91 Ala94 Val95 Asp98 Phe124 Lys127 Trp128 Ile131 Leu32 Ala139	Asn96 Val99 Phe100 Ile103 Ile104 Ala154 Tyr157

Underlined and bold residues correlate with putative thymoquinone and/or substrate binding site of the enzyme.

### The impact of thymoquinone on the Ax4 proliferation, GST activity, and expression

**Fig 2:**
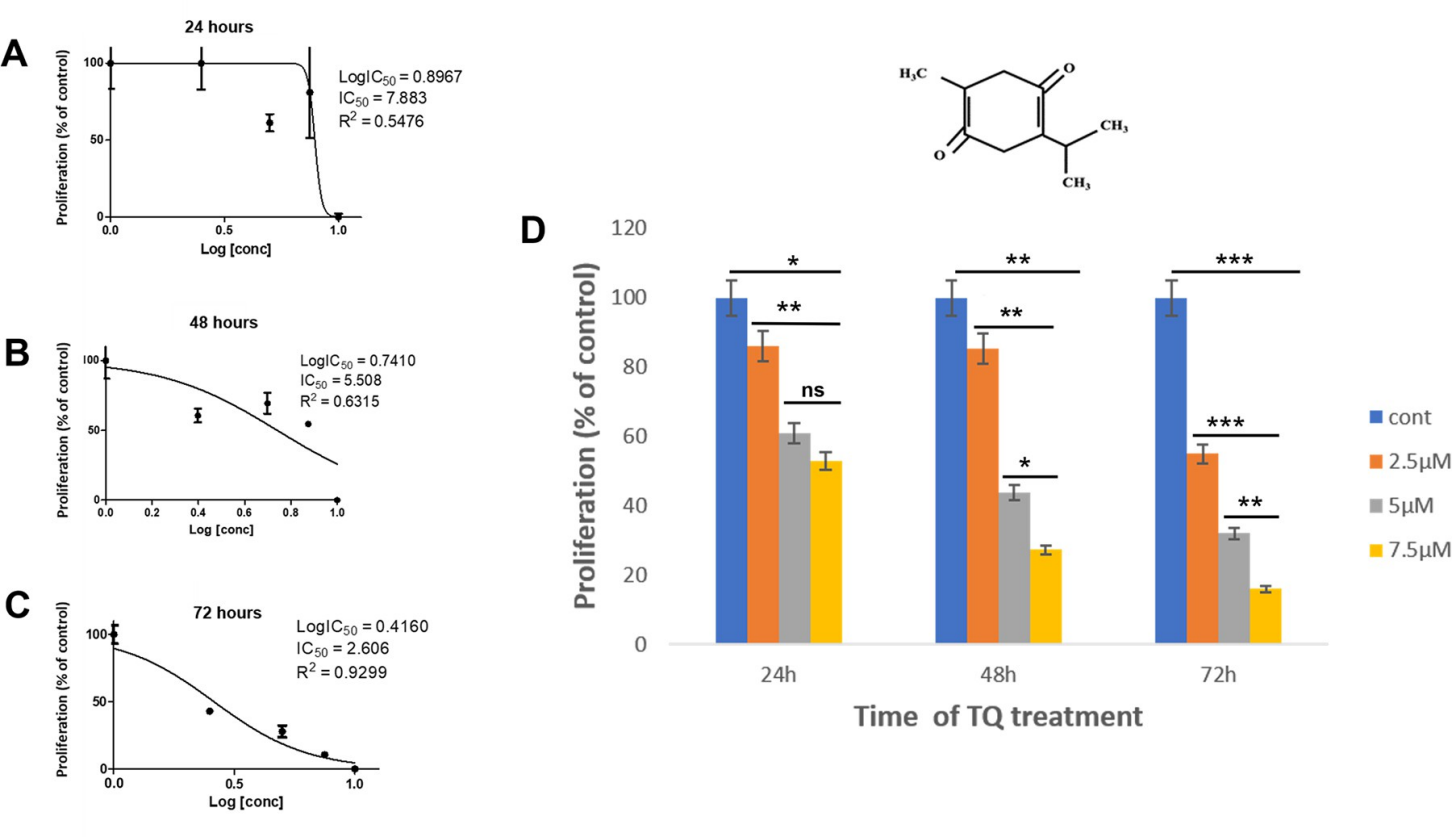
The impact of thymoquinone (TQ) on *Dictyostelium* Ax4 (axenic cells) survival and proliferation. (A-C), Dose response curves of AX4 cell proliferation in the absence (control) or presence of various concentrations (see Methods) of thymoquinone after 24 hr (A) 48 hr (B) and 72 hr to determine inhibitory concentration (IC50) on proliferation in treated cells vs. controls (100%). D), Histograms demonstrate the effect of 2.5, 5.0, and 7.5uM thymoquinone to inhibit proliferation in a dose-dependent manner during 72hr period. Trypan blue assays were used to determine cell viability. The data reflect the analysis of three independent experiments and is expressed as the standard error of the mean (SEM); p values were calculated using Student’s t-test (*< 0.05).

The analysis of *in silico* studies established a basis to examine the impact of thymoquinone on *D*. *discoideum in vivo*. Axenic (AX4) amoebae were grown in shaking culture with different concentrations of thymoquinone (0.25, 0.5, 1, 2.5, 5.0, 7.5, and 10μM) for 72hr. An IC50 (proliferation compared to controls) of 7.883 μM thymoquinone was determined after 24hr of growth (Fig 2A), while amoebae were able to tolerate and maintain viability at a maximal concentration of 7.5μM during this period. The concentrations of thymoquinone for subsequent experiments were established at 2.5, 5.0, and 7.5uM. Assessment at 2.5, 5.0, and 7.5μM thymoquinone showed that at 24hr, proliferation was reduced by 24%, 41%, and 47%, respectively, when compared to controls. Additionally, thymoquinone treatment exhibited a dose-dependent dynamic that negatively impacted proliferation at 48hr, with its most significant effect at 7.5μM at 72hr of treatment (Fig 2D).

**Fig 3:**
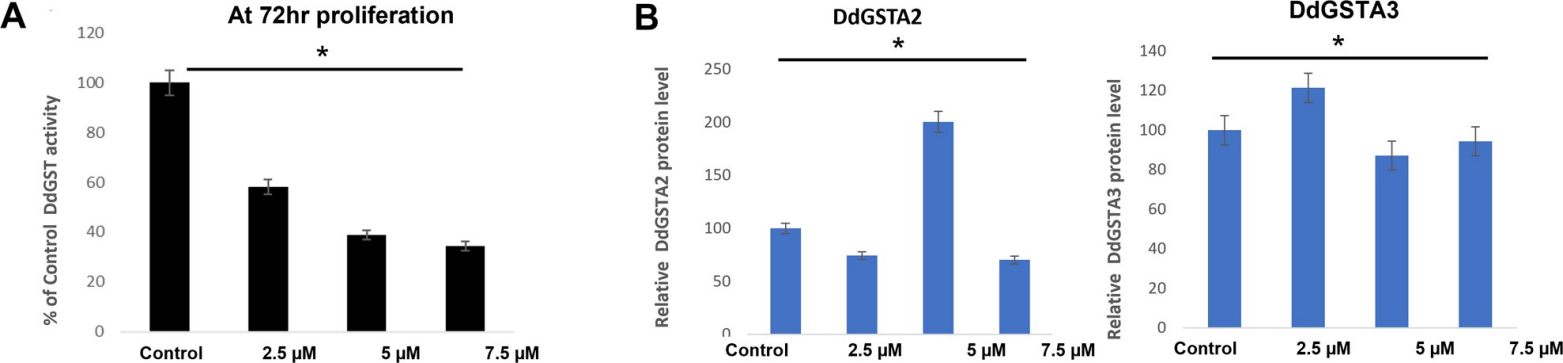
Impact of thymoquinone (72 hr) on cytosolic DdGST enzyme activity and DdGSTA2, DdGSTA3 expression in vegetative amoeba. (A), Cytosolic DdGSTs activity (CDNB conjugation) was inhibited by thymoquinone at all concentrations, exhibiting more than 50% inhibition at 7.5μM. (B) ELISA measurement of DdGSTA2 and DdGSTA3 expression levels in vegetative amoeba showed that thymoquinone generally reduces expression of both isozymes; in contrast, thymoquinone increased DdGSTA2 expression at 5μM and DdGSTA3 isozyme at 2.5μM when compared to controls. Data from three independent experiments is expressed as the standard error of the mean (SEM) and p values were calculated using Student’s t-test (*< 0.05).

In proliferating Ax4 cells, *Dictyostelium* GST (DdGST) enzymes were responsive to thymoquinone, as cytosolic DdGST activity was reduced by 41.8%, 61.1%, and 65.6%, respectively, at 2.5, 5.0, and 7.5μM (Fig 3A). The reduction in cytosolic DdGST activity correlated with the decrease in cell proliferation relative to thymoquinone concentration. Previous work from our laboratory suggests that *gstA2* and *gstA3* regulation plays important roles in *Dictyostelium* growth and development [26]. The effect of thymoquinone on DdGSTA2 and DdGSTA3 expression revealed differential effects on each isozyme pertaining to thymoquinone concentration. While significant, reduced expression of DdGSTA2 (at 2.5μM and 7.5μM thymoquinone) and DdGSTA3 (at 5.0μM and 7.5μM thymoquinone) occurred at 72hr, significant increases were observed that were isozyme-specific at different concentrations (DdGSTA2 at 5.0μM; DdGSTA3 at 2.5μM), with DdGSTA2 exhibiting a two-fold increase in expression when compared to controls (Fig 3B).

**Fig 4:**
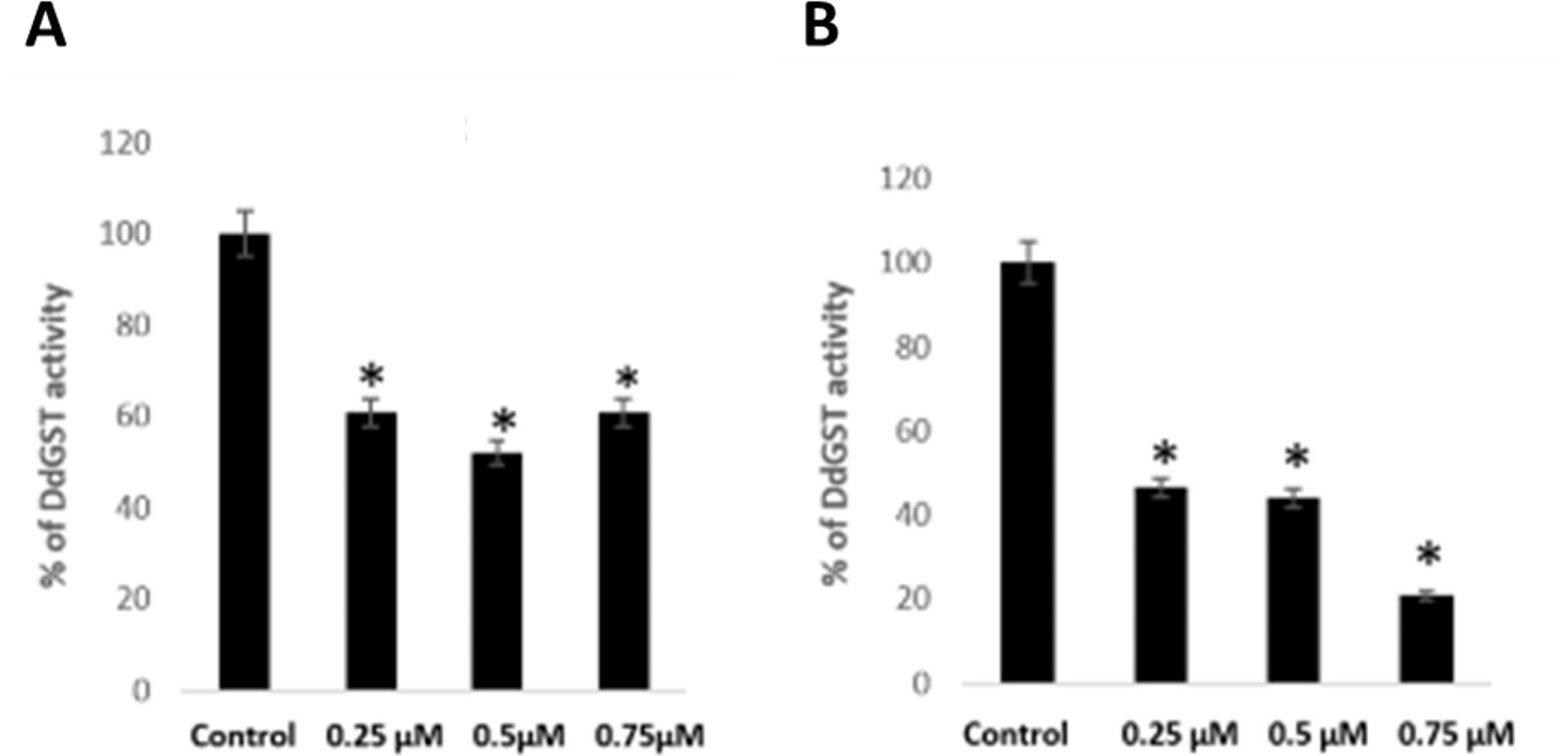
Impact of thymoquinone on recombinant DdGSTA2 and DdGSTA3 isozyme activity *in vitro*. CDNB assays conducted with (A) rDdGSTA2 and (B) rDdGSTA3 isozymes demonstrate thymoquinone effectively reduces the amount of measurable GS-CDNB conjugate at 0.25μM, 0.5μM, 0.75μM in comparison to controls. Data from three independent experiments are expressed as the standard error of the mean (SEM) and p values were calculated using Student’s t-test (*< 0.05).

These observations established a basis to determine the effect of thymoquinone on enzymatic activity (CDNB assays) of purified, recombinant DdGSTA2 and DdGSTA3 isozymes *in vitro*. For rDdGSTA2, we found that 0.25μM, 0.5μM, and 0.75μM thymoquinone reduced the amount of measurable GS-DNB conjugate (61%, 55%, 61%, respectively), and similar profiles were observed with respect to rDdGSTA2 (53.5%, 56%, 79.1%, respectively) when compared to untreated amoebae (Fig 4A and 4B). These findings correlate with the molecular modeling and proliferation studies, suggesting that thymoquinone negatively inhibits the DdGSTA2 and DdGSTA3 activity by direct binding, and may interfere with *D*. *discoideum* growth and development.

### Thymoquinone alters *D*. *discoideum* development, morphogenetic markers, and GST enzymes during starvation

**Fig 5:**
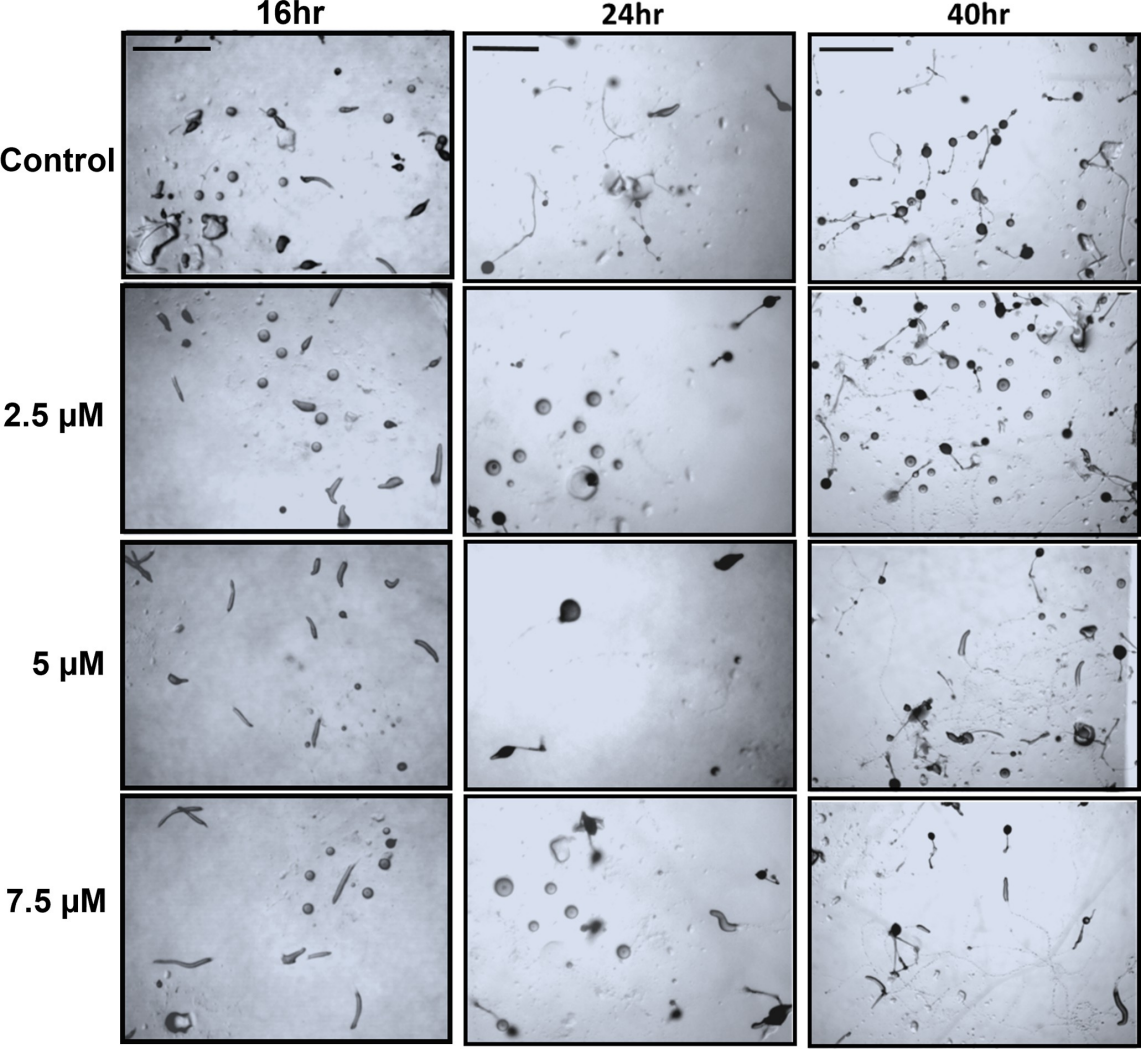
Effect of thymoquinone on development of starved *D*. *discoideum* amoebae. 6hr starved amoeba were aliquoted onto non-nutrient agar without (controls) or with 2.5uM, 5uM, or 7.5uM thymoquinone. Cells were imaged at the indicated times with stereomicroscope. Images are representative of observations from at least five aliquots (spottings) of three independent experiments. Delayed morphogenesis, abnormal development, and reduced numbers of fruiting bodies are a function of the increased concentration of thymoquinone. Scale bar = 1 mm.

To determine the effect of thymoquinone on the *D*. *discoideum* development and morphogenesis, vegetative amoebae were starved, and developed in the presence of 2.5μM, 5.0μM, and 7.5μM thymoquinone. Untreated starved amoebae progressed through normal stages of developmental timing, and morphogenesis, culminating in robust, numerous fruiting bodies after 24hr. Consistent with the effects of thymoquinone at early-stage development, we observed characteristic features of abnormal development regarding the size, altered morphology, and a reduced number of fruiting bodies, which occurred in a dose-dependent manner (Fig 5). This suggested that thymoquinone effects critical periods of early development that may involve transition from feeding behavior to starvation and morphogenesis.

**Fig 6:**
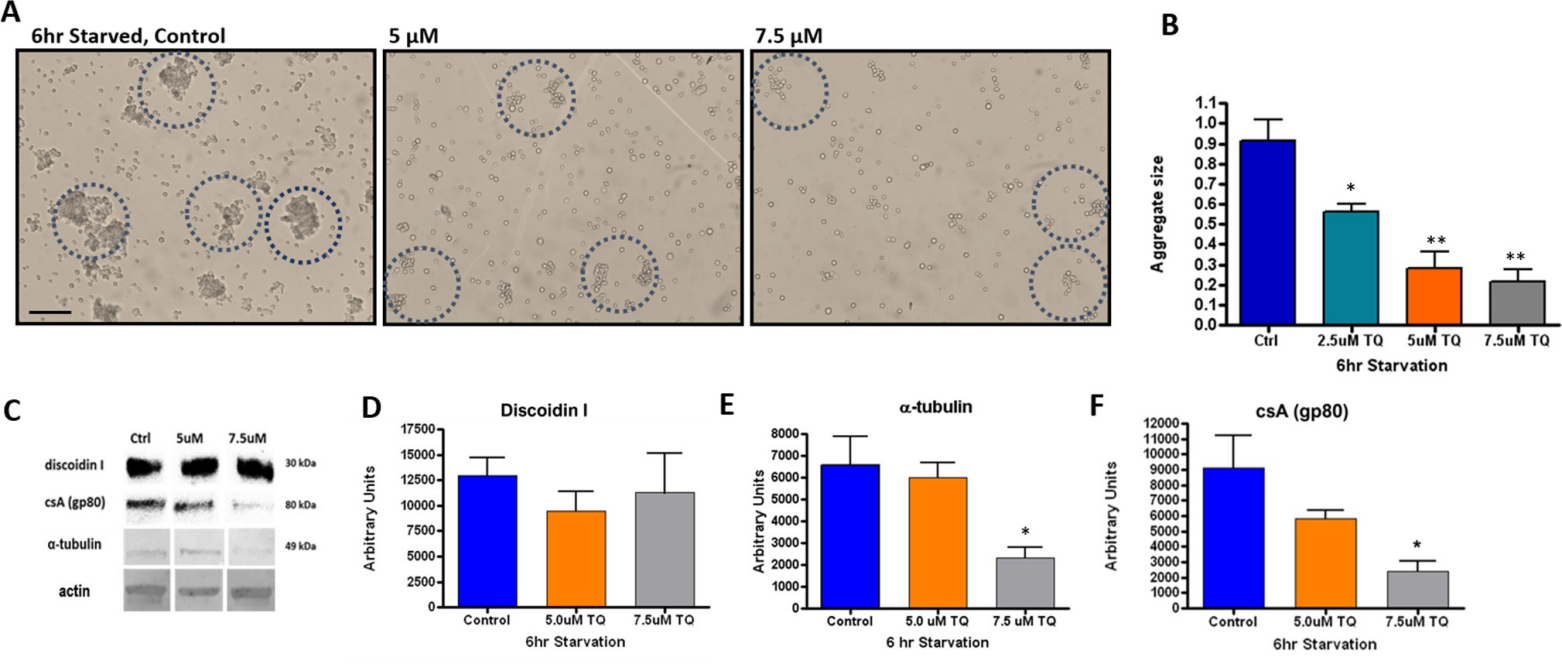
Impact of thymoquinone on *Dictyostelium* aggregation and developmental markers during starvation-induced development. A, B) In the absence of thymoquinone, starvation-induced control amoebae readily assembled into large, multicellular aggregates (hashed circles) within the 6hr examination period. Scale bar = 80 microns. Thymoquinone significantly inhibited formation of robust aggregates in a dose-dependent manner (B). C), Representative immunoblots of discoidin I, csA(gp80), α-tubulin expression in the absence or presence of 5μM and 7.5μM thymoquinone. Histograms (B, D-F) represent the standard error of the mean of three independent experiments and p values were calculated using one way ANOVA, followed by Tukey’s post hoc test for multiple comparisons (***< 0.001).

The results of 24hr development suggested that thymoquinone may act at early or intermediate stages of starvation. To determine this, we starved amoebae for 5-6hr in the presence of 2.5μM, 5.0μM, and 7.5μM thymoquinone. Untreated amoebae readily aggregated and displayed large clusters that comprised multiple cells. In contrast, amoebae starved in 2.5μM thymoquinone formed few aggregates, and were generally smaller in size when compared to controls. 7.5μM thymoquinone markedly restricted cell aggregation to roughly two to four cells (Fig 6A). Next, we examined the expression of various markers such as discoidin I, tubulin, and contact site A (gp80), which are essential for starving amoebae to initiate cell-cell communication, establish polarity, and promote chemotaxis and aggregation [31]. Expression of the prestarvation lectin discoidin I was measured, with small, insignificant reduction in expression at 5μM and 7.5 μM thymoquinone (Fig 6C and 6D). In contrast, thymoquinone significantly reduced α-tubulin and csA(gp80) expression at 5μM (60.6%, 38.9% respectively) and 7.5μM (78.95%, 72.3%, respectively) when compared to untreated controls (Fig 6D–6F).

**Fig 7:**
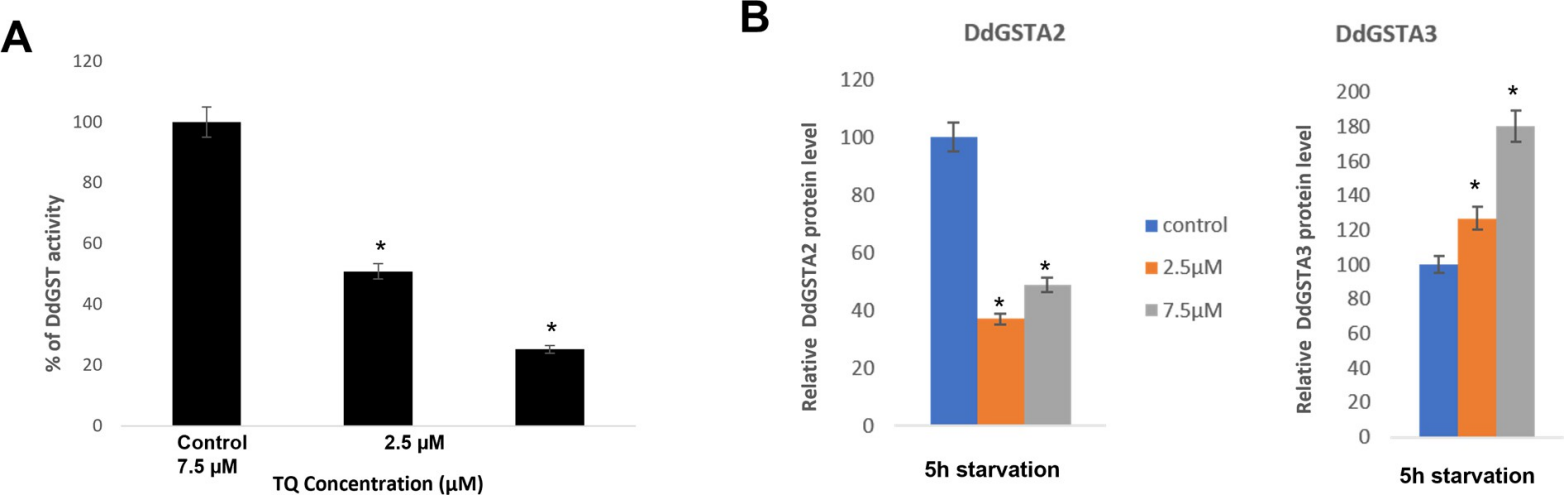
Impact of thymoquinone on cytosolic DdGST enzyme activity and DdGSTA2, DdGSTA3 isozymes expression in 5hr starved amoebae. (A), Measurable CDNB conjugation was reduced by the presence of thymoquinone was dose-dependent, with the greatest effect (more than 50% inhibition) at 7.5μM. (B), ELISA analysis of DdGSTA2 and DdGSTA3 content in starved amoeba revealed that thymoquinone negatively impacted expression of DdGSTA2; in contrast, there was increased expression of DdGSTA3 when compared DdGSTA2 and untreated controls. Data are expressed as the standard error of the mean (SEM) of three independent experiments and p values were calculated using Student’s t-test (*< 0.05).

Cytosolic DdGST activity in 5hr starved amoeba was significantly reduced under 2.5μM and 7.5μM thymoquinone treatment (50.87% and 15.28%, respectively) in comparison to control, untreated amoebae (Fig 7A). When endogenous DdGSTA2 and DdGSTA3 expression was examined at 2.5 and 7.5μM thymoquinone we found that the levels were decreased by 37% and 48.9% (respectively) when compared to controls (Fig 7B). In striking contrast, 2.5μM and 7.5μM increased DdGSTA3 expression by 27% and 80%, respectively, when compared to controls (Fig 7B).

### Thymoquinone, reactive oxygen species (ROS) and GSH content in starved cells

**Fig 8:**
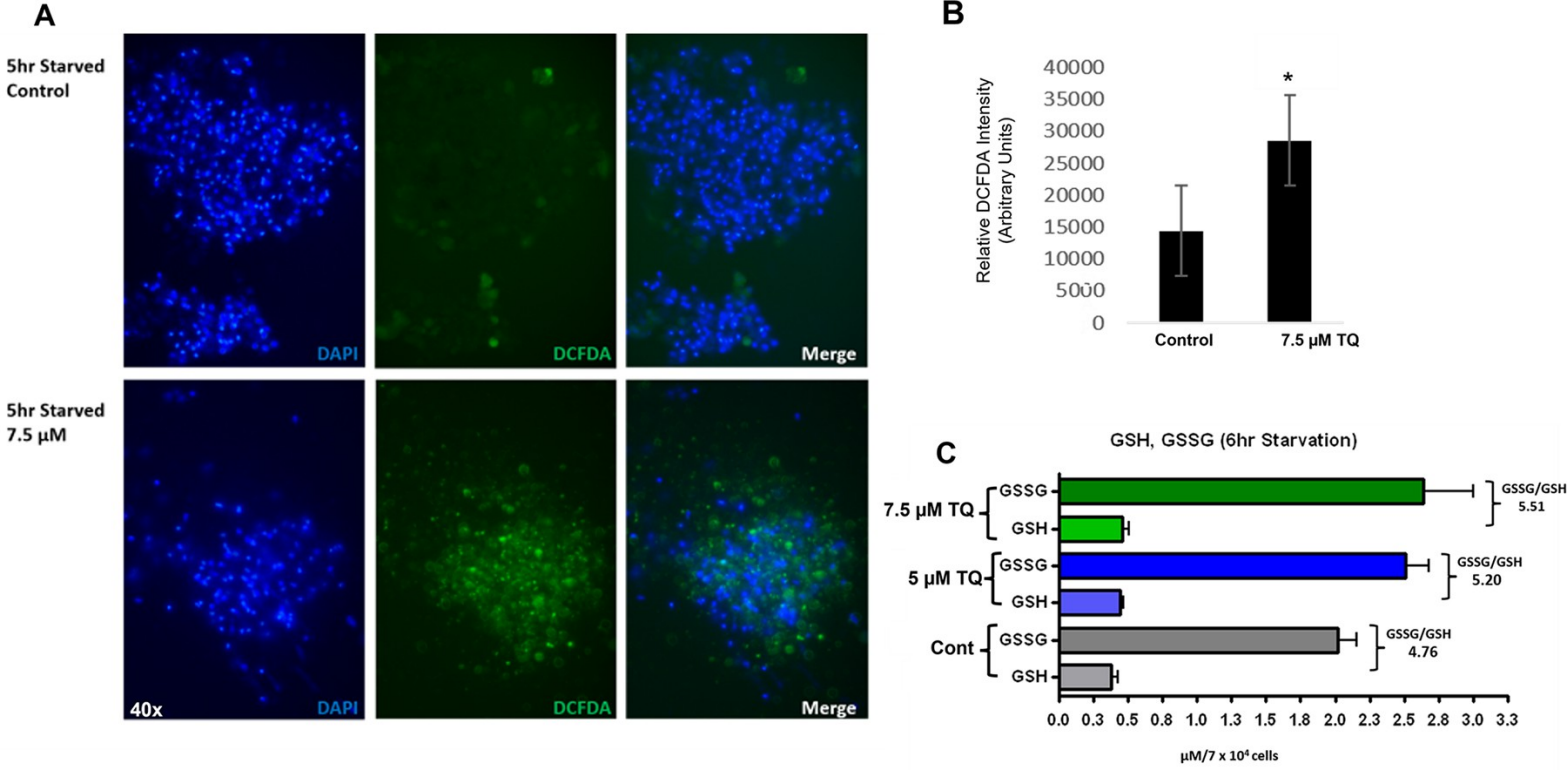
Measurement of reactive oxygen species (ROS) and GSH content in 5hr starved amoebae treated with thymoquinone (TQ). (A) Consistent with previous observations, controls exhibited robust adhesion and clustered aggregates in comparison to fewer aggregates in the presence of thymoquinone (7.5μM). DCFDA-ROS fluorescence intensity (green) in controls revealed basal intensity of cells associated with ROS. In contrast, thymoquinone significantly increased production of DCFDA in cells when compared to controls. DAPI nuclear staining is identified in blue. (C), Analysis of reduced (GSH) and oxidized (GSSH) glutathione content was slightly elevated in the presence of thymoquinone. Data in (B) are presented as the standard error of the mean (SEM) from three independent experiments and p values were calculated using Student’s t-test (*< 0.05). Data in (C) is expressed as the standard error of the mean (SEM) of three independent experiments and p values were calculated using a one-way ANOVA and is not significant.

The regulation of glutathione (GSH) and reactive oxygen species (ROS) are essential to early stage aggregation and development of *D*. *discoideum* [32, 33]. Because thymoquinone disrupted *D*. *discoideum* development, cytosolic activity, and expression of individual DdGST isozymes (Fig 7A and 7B), DCFDA was used to measure the level of ROS in response to thymoquinone during early-stage starvation. A notable, two-fold increase in ROS was observed in cells that were treated with 7.5 μM thymoquinone for 5hr (Fig 8A and 8B). Whereas GSH is known to control ROS production and is implicated in thymoquinone resistance [34], recent findings indicate that sulfur/cysteine sequestration is a rate limiting variable to initiate development during starvation [33]. Moreover, because GSH is an essential to the conjugation of multiple substrates in GST enzyme activity, we measured the levels of oxidized and reduced glutathione (GSSG, GSH). In contrast to the changes in ROS, thymoquinone did not significantly alter GSSG/GSH ratios, as the levels were comparable to that of controls (Fig 8C).

## Discussion

The life cycle of *D*. *discoideum* involves complex gene regulatory events that coordinate signaling processes during its development and morphogenesis. Due to its anti-proliferative and anti-inflammatory properties, thymoquinone has received an increased *focus because of its* potential health benefits related to cancer [35], autoimmunity [36], metabolic disorder [37], as it also promotes anti-oxidant mechanisms [38]. Thymoquinone resides within a class of phytochemicals and polyphenols that modulate the activity and expression of glutathione-dependent enzymes in eukaryotic cells and tissues [39]. Previously, it was reported that thymoquinone and curcumin inhibition of GSTs and GSH-dependent enzymes in the parasite, *Fasciola gigantica* correlated with oxidative stress and cellular apoptosis [40]. Studies from our laboratory also demonstrate the negative effect of curcumin on *D*. *discoideum* GSTs, resulting in delayed growth and development in the organism [10]. However, little is known about the mechanism by which thymoquinone-mediated effects on GSH-dependent enzyme function in cellular proliferation, development, and differentiation in *Dictyostelium*.

*In silico* modeling between thymoquinone and DdGSTA2 and DdGSTA3 isozymes suggests that it binds within the substrate (H domain) of each isozyme. Whereas only a few thymoquinone-GST modeling studies are published [40], the strong affinity of thymoquinone for catalytic centers of the GST enzyme remains consistent across different classes (alpha or sigma) of isozymes [40]. This, coupled to its potential to bind at non-catalytic sites suggests that thymoquinone alters GST function directly in many ways that impact dimerization, conjugation, impacting other non-enzymatic capacities of the protein. Additionally, the apparent reduction of rDdGSTA2 and rDdGSTA3 activity in CDNB assays must be interpreted critically. To be sure, thymoquinone may bind to factors in the CDNB assay such as GSH [41], CDNB, individual GST isozymes, as well as post-conjugation entities such as the spectrophotometrically measured product, GS-DNB (glutathionyl dinitrobenzene). Thus, interpretations of GST activity inhibition by thymoquinone may be oversimplified due to the complexity of thymoquinone interaction with many biochemical factors. The ability of thymoquinone to inhibit total GST activity in proliferating and developing amoebae in a dose-dependent manner may reflect its capacity for strong binding to the H-site motifs across all (α1-α5) DdGSTAs *in vivo*. In this vein, we hypothesize that such binding may compete for GSH, or interfere with substrate binding of one or more of the DdGSTA isozymes, impacting endogenous antioxidant capacity and substrate conversion *in vivo*. How precisely *in vitro* assays of thymoquinone-GST inhibition correlated with *in vivo* findings is likely complicated by other endogenous factors that may be targets for thymoquinone [42–44].

Previous studies from our laboratory demonstrated the anti-proliferative effect of another polyphenol, curcumin, which was accompanied by altered GSTs activity and *gstA2* and *gstA3* transcription in vegetative amoeba [10]. With an IC50 of 7.8 μM in *D*. *discoideum*, we found that proliferation was inhibited significantly by 2.5 μM thymoquinone, a concentration that is ten-fold less than effective doses in cancer cell lines [45]. Here, the anti-proliferative effects of thymoquinone in vegetative amoeba was coupled to inhibition cytosolic GST activity and effectively reduced expression of DdGSTA2 and DdGSTA3 isozymes across most concentrations. In mammalian cells, GSTs interact with important signaling factors that regulate the cell cycle [46], and heightened GST activity and expression is associated with resistance to chemotherapy, proliferation and migration [47]. El-najjar and colleagues demonstrated that thymoquinone-induced apoptosis of cancer cells is associated with reactive oxygen species (ROS)-mediated phosphorylation of (MAPK) JNK and ERK signaling [48], and that N-acetyl cysteine (NAC) abolishes this effect; this suggests that NAC confers antioxidant (anti-apoptotic) potential to cells in the form of GSH derivatives. Indeed, GSH depletion leads to growth arrest and induces apoptosis in many microorganisms [49]. Strong evidence reveals that GSTA enzymes directly bind to, or conjugate GSH to endogenous factors that mediate cell cycle control, survival, and apoptosis eukaryotic cells [46]. The relationship between DdGSTA enzyme function and MAPK/ERK signaling in the life cycle of *D*. *discoideum* [50] remains to be elucidated. Given that *D*. *discoideum* expresses only alpha class isozymes, the influence of polyphenols such as curcumin and thymoquinone present new opportunities to identify unique and exclusive roles of DdGSTA functions within and beyond this organism.

When *D*. *discoideum* vegetative cells are starved, synthesis and secretion of cAMP promotes chemotaxis, aggregation, and stage-specific morphogenesis to form multicellular fruiting bodies [51]. This process is initiated by production and utilization of reactive oxygen, which is governed by GSH regulation that promotes cAMP signaling and development [52]. Thymoquinone exerts multiple, differing effects across many cell lines, generate ROS, and reduce cell membrane integrity [53]. Alternatively, thymoquinone can exhibits pro-oxidant (semiquinone) [54] properties that are unique to the cellular microenvironment [55]. Also, the depletion of GSH alters redox balance, resulting in an increase in the ROS [56, 57]. In *Dictyostelium*, recent studies have reinforced the importance of redox balance, particularly the ROS-mediated sequestration of cysteine (of tripeptide GSH) as critical determinates of cell fate in *D*. *discoideum* development [33]. The finding that thymoquinone induced ROS (DCFDA staining) without disruption to GSH levels (Fig 8C), suggests that its inhibitory effect on *D*. *discoideum* development is complex, and may directly or indirectly influence targets beyond GSH-synthesizing enzymes.

Previously, we reported that siRNA knockdown of DdGSTA2 levels reduced aggregation and morphogenesis, implicating a role for this isozyme in development. Similarly, inhibition of total DdGSTA activity amid GSSG/GSH stability suggests that reduced catalytic function of DdGSTA impacts GSH- or GST-dependent developmental signaling pathways. The studies herein provide evidence for the direct interaction of thymoquinone with GST enzymes of the *D*. *discoideum*. The ability of thymoquinone to inhibit GST function within the unicellular and multicellular stages of this eukaryotic developmental model, is foundational to a greater understanding of the therapeutic efficacy of polyphenols in mammalian cells.

## Supporting information

S1 Data (DOCX)

S1 File (DOCX)
