# Supplementary material for: Thymoquinone effect on the Dictyostelium discoideum model correlates with functional roles for glutathione S-transferases in eukaryotic proliferation, chemotaxis, and development
Source: PLoS One. 2023 Mar 1;18(3):e0282399. doi: 10.1371/journal.pone.0282399 (PMC9977050; doi:10.1371/journal.pone.0282399)
Supplement: S1 Data — (DOCX) [file pone.0282399.s001.docx]

Supplementary Data

Table 1: 18 possible binding sites between DdGSTA2 and thymoquinone.

Table 2: 14 possible binding sites between DdGSTA3 and thymoquinone.
