# Supplementary figures and images for: Thymoquinone effect on the Dictyostelium discoideum model correlates with functional roles for glutathione S-transferases in eukaryotic proliferation, chemotaxis, and development
Source: PLoS One. 2023 Mar 1;18(3):e0282399. doi: 10.1371/journal.pone.0282399 (PMC9977050; doi:10.1371/journal.pone.0282399)

CSA, Discoidin


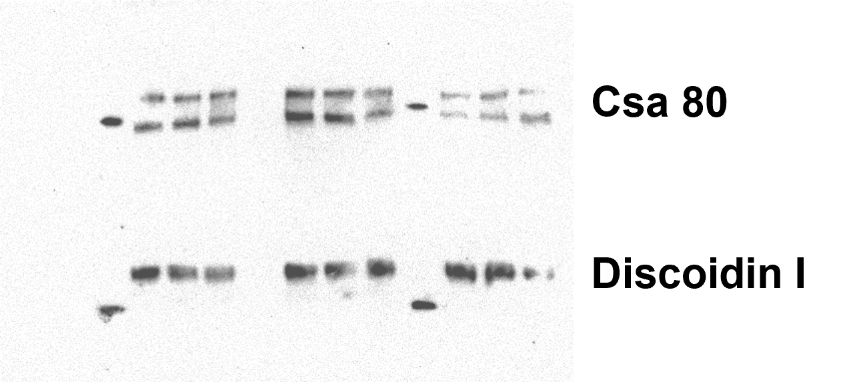


5uM tq ctrl tq 7.5uM tq

ACTIN


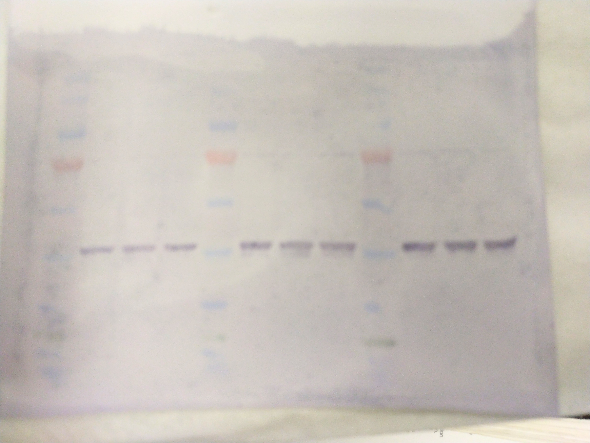


5uM tq ctrl tq 7.5uM tq

TUBULIN


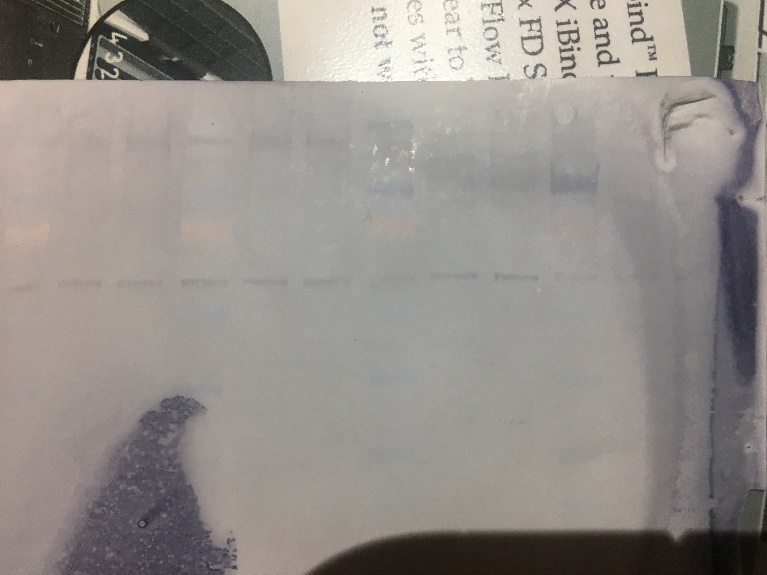


5uM tq ctrl tq 7.5uM tq

Supplement: S1 File — (DOCX) [file pone.0282399.s002.docx]
